# Langerhan’s cell histiocytosis: A single institutional experience

**DOI:** 10.4103/0971-5851.71655

**Published:** 2010

**Authors:** Tejinder Singh, C. T. Satheesh, L. Appaji, B. S. Aruna Kumari, H. S. Mamatha, G. V. Giri, Clementina Rama Rao

**Affiliations:** *Department of Medical Oncology, Kidwai Memorial Institute of Oncology, Bangalore - 560 030, India*; 1 *Department of Pediatrics Oncology, Kidwai Memorial Institute of Oncology, Bangalore - 560 030, India*; 2 *Department of Radiation Oncology, Kidwai Memorial Institute of Oncology, Bangalore - 560 030, India*; 3 *Department of Pathology, Kidwai Memorial Institute of Oncology, Bangalore - 560 030, India*

**Keywords:** *Bone lesion*, *chemotherapy*, *Langerhan’s cell histiocytosis*

## Abstract

**Background:**

Langerhans cell histiocytosis (LCH) is a disease that primarily affects bone but can be associated with a clinical spectrum that ranges from a solitary bone lesion with a favorable natural history to a multisystem, life-threatening disease process.

**Aim:**

We analyzed our single institutional experience of managing children with LCH.

**Settings and Design:**

A total of 40 children of LCH, managed in tertiary cancer center in South India in the period from 2001 to 2005, were evaluated retrospectively.

**Materials and Methods:**

Clinicopathological features, laboratory findings, treatment modalities and long-term outcome were analyzed.

**Results:**

Children were aged between 2 months and 12 years, with a mean of 3 years. Majority of the children were below 5 years of age. Group B constituted a bulk of children. Disseminated cases were less (five patients). Liver function dysfunction was seen in four (10%) children. Pulmonary interstitial infiltrates were seen in two (5%) cases. Diabetes insipidus manifested in three patients. There was one death.

**Conclusion:**

A better understanding of the etiology and pathogenesis of LCH will result in more directed and efficacious treatment regimens.

## INTRODUCTION

The diagnosis of Langerhan’s cell histiocytosis (LCH) is often difficult and delayed.[1] The following organs are commonly involved in LCH: bone (pelvis, femur, ribs, skull, and orbit), skin, lymph nodes, bone marrow, lungs, hypothalamic pituitary axis, spleen and liver. Bone involvement with or without other associated sites is the most common manifestation of LCH and has been observed in 80-100% of cases based on a review of the literature. Clinical manifestations depend on the site of lesions, number of involved sites and the extent to which the function of the involved organs is compromised. The course of the disease varies from spontaneous resolution to a progressive multisystem disorder with organ dysfunction and potential life-threatening complications.[2–4]

The annual incidence of LCH is reported to be 0.5–5.4 million children per year.[5,6] Males are affected to a greater degree than females. It is a disease of childhood, with more than 50% of cases diagnosed between 1 and 15 years. There is peak in the incidence between 1 and 4 years. Although diagnosis often occurs in childhood, many cases of childhood onset progress into adult life. We analyzed our single institutional experience of managing children with LCH.

## MATERIALS AND METHODS

A total of 40 children of LCH, managed in tertiary cancer center in South India in the period from 2001 to 2005, were evaluated retrospectively for clinicopathological features, laboratory findings, treatment modalities and long-term outcome. Presumptic diagnosis based on morphologic characteristics was used in a majority of them and supplemental stains for S-100 protein/CD 1a were performed for definitive/designated diagnosis. The extent of the disease was classified into three groups – Group A: patients with lesion in multiple bones or more than two lesions in one bone; Group B: patients with soft tissue involvement with or without bony lesion (lymphadenopathy, diabetes insipidus or growth hormone deficiency syndrome); and Group C: patients with multisystem disease with organ dysfunction (liver, lung and/or bone marrow involvement).[7]

Children with Group A disease were treated with prednisolone 40 mg/m^2^/day for 4 weeks and then the drug was tapered over 2 weeks. They also received vinblastine 6 mg/m^2^/day IV bolus on days 1, 8, 15, 22, 29 and 36. Children with Group B and C disease received treatment according to DAL HX-83 protocol[8] which consisted of drugs like prednisolone, vinblastine, etoposide and 6-mercaptopurine (6-MP).

## RESULTS

Children were aged between 2 months and 12 years, with a mean of 3 years. The M:F ratio was 3:1. Group A disease was seen in 15 children. Group B disease was seen in 20, whilst multisystem involvement with an evidence of organ dysfunction (Group C) was seen in 5 children.

Signs/symptoms and bone lesion distribution are summarized in Tables 1 and 2, respectively. Liver function dysfunction was seen in four (10%) children. Bone marrow involvement was not seen in any patient. Pulmonary interstitial infiltrates were seen in two (5%) cases. Diabetes insipidus manifested in two patients at presentation and developed in one child after 6 months. The mean follow-up duration was 18 months.

**Table 1:** Signs/symptoms of LCH

Signs/symptoms	No. (%)
Bone involvement	28 (70)
Lymphadenopathy	16 (40)
Fever	16 (40)
Skin involvement	10 (25)
Ear discharge	4 (10)
Diabetes insipidus	3 (7.5)
Jaundice	3 (7.5)
Paraparesis	1 (2.5)
Loosening of teeth	2 (5)

**Table 2:** Bone lesion distribution in LCH

Bone involved	No. (%)
Skull vault	28 (70)
Lower limb bones	8 (10)
Pelvis	4 (10)
Vertebrae	2 (5)
Mandible/maxilla	2 (5)

Group A disease (*n*=15): 14 children (93.3%) had a complete response. Recurrence occurred in one patient who subsequently defaulted.

Group B disease (*n*=20): Disease free survival without recurrences was seen in 12 children (60%). Three patients had recurrence and subsequently defaulted. Five families refused therapy.

Group C disease (*n*=5): Out of the five patients, two defaulted. Three patients received treatment. One of them had a complete response without recurrences. The second patient relapsed and was treated again and is disease free since then. Third patient died because of progressive disease.

## DISCUSSION

Children with LCH may present with clinical presentations involving a wide spectrum of osseous and extraosseous manifestations. Diagnosis of LCH can be presumptive, designated and definitive.[9–11] Presumptive diagnosis is based on light morphologic characteristics. Designated diagnosis is based on light morphologic features plus two or more supplemental stains for adenosine triphosphatase, S-100 protein, α-D-mannosidase and peanut lectin. A definitive diagnosis requires the immunohistochemical identification of the presence of Langerhans cells by cell surface CD1a or is made by the presence of cells with Birbeck granules by electron microscopy. The CD1a surface antigen can now be identified routinely from paraffin-embedded specimens; thus, electron microscopy is only rarely required.

Our study is a retrospective analysis of 40 children with LCH, managed in tertiary cancer center in South India in the period from 2001 to 2005. Every year, on average, seven to eight children are diagnosed Swith LCH in our institute. Age distribution and sex distribution in the study followed the typical distribution described in literature.[1,12,13] Majority of the children were below 5 years. Group B constituted a bulk of children. Disseminated cases were less and most of the children were younger than 2 years in this group.

Unusual sites of bone involvement observed included zygomatic bone, scapula and sternum. Clinical signs and symptoms in our study had largely mimicked earlier reports. Our experience of 40 cases, although less in number, reaffirms that chemotherapy is curative and these patients can be managed without any lifethreatening complication. The prognosis for patients with Group A and B is fairly good with chemotherapy. However, new therapies are required for patients with Group C as the outcome remains poor.
